# Evaluation of Memory Enhancing Clinically Available Standardized Extract of *Bacopa monniera* on P-Glycoprotein and Cytochrome P450 3A in *Sprague-Dawley* Rats

**DOI:** 10.1371/journal.pone.0072517

**Published:** 2013-08-28

**Authors:** Rajbir Singh, Jagadeesh Panduri, Devendra Kumar, Deepak Kumar, Hardik Chandsana, Rachumallu Ramakrishna, Rabi Sankar Bhatta

**Affiliations:** 1 Pharmacokinetics and Metabolism Division. CSIR-Central Drug Research Institute, Lucknow, Uttar Pradesh, India; 2 National Institute of Pharmaceutical Education and Research, Raibarelly, Uttar Pradesh, India; University of South Alabama Mitchell Cancer Institute, United States of America

## Abstract

*Bacopa monniera* is a traditional Ayurvedic herbal medicine used to treat various mental ailments from ancient times. Recently, chemically standardized alcoholic extract of *Bacopa monniera* (BM) has been developed and currently available as over the counter herbal remedy for memory enhancement in children and adults. However, the consumption of herbal drugs has been reported to alter the expression of drug metabolizing enzymes and membrane transporters. Present study in male *Sprague-Dawley* rat was performed to evaluate the effect of memory enhancing standardized extract of BM on hepatic and intestinal cytochrome P450 3A and P-glycoprotein expression and activity. The BM (31 mg/kg/day) was orally administered for one week in BM pre-treated group while the control group received the same amount of vehicle for the same time period. The BM treatment decreased the cytochrome P450 3A (CYP3A) mediated testosterone 6β-hydroxylation activity of the liver and intestine by 2 and 1.5 fold, respectively compared to vehicle treated control. Similarly pretreatment with BM extract decreased the expression of intestinal P-glycoprotein (Pgp) as confirmed by Western blot analysis but did not alter the expression of hepatic Pgp. To investigate whether this BM pretreatment mediated decrease in activity of CYP3A and Pgp would account for the alteration of respective substrate or not, pharmacokinetic study with carbamazepine and digoxin was performed in BM pre-treated rats and vehicle treated rats. Carbamazepine and digoxin were used as CYP3A and Pgp probe drugs, respectively. Significant increase in AUC and Cmax of carbamazepine (4 and 1.8 fold) and digoxin (1.3 and 1.2 fold), respectively following the BM pre-treatment confirmed the down regulation of CYP3A and Pgp.

## Introduction

*Bacopa monniera* (BM) is used traditionally in Ayurvedic medicine to improve memory and intellect. Pharmacologically active ethanolic extract of BM has been reported to contain a mixture of triterpenoid saponins designated as bacosides A and B as main active constituents [1], [2]. Recently, a chemically standardized alcoholic extract of *Bacopa monniera* (BM) has now been made available for clinical use by the CSIR-Central Drug Research Institute in India after clinical trials in human volunteers [3]. This standardized extract of BM called BESEB (Bacoside Enriched Standardized Extract of Bacopa) is now available over-the- counter as a memory enhancing herbal preparation (http://www.cdriindia.org/Memory_Sure.pdf,). Clinical studies suggest that regular administration of BM (300 mg) for 5 to12 weeks improves the speed of visual processing, learning rate, memory consolidation [4] and decreases the rate of forgetting of newly acquired information in healthy individuals [5]. Although we used standardized marketed formulation of *Bacopa monniera* contains 50±5% of bacoside A and bacoside B but the recent studies on *Bacopa monniera* have revealed that bacosides A and B are not single chemical entities as reported, but they are found to be mixtures of saponins [6]. Bacoside A chemical composition has recently been established as a mixture of four triglycosidic saponins, Bacoside A3,Bacopaside II, jujubogenin and Bacopasaponin C [7] however no reports are available on the chemical composition of bacoside B. Moreover, with increased consumption of herbs the herb-drug interactions are becoming the major concern. Additionally, herbs are generally orally consumed and with a high concentration in gut lumen they exert major effects on the gastrointestinal tract. Furthermore, with people’s view that natural products are safe, herbal drug consumption has been increased in recent years and are often co-administered with therapeutic drugs that alleviates the potential of herb-drug interactions [8], [9].

Liver and intestine contains various drug metabolizing enzymes and membrane transporters that work as major metabolic barrier to oral drug administration. Among various gastrointestinal drug metabolizing enzymes and membrane transporters, cytochrome P450 (CYP) mediated metabolism and P-glycoprotein (Pgp) mediated efflux play important role in influencing the oral bioavailability of respective substrates drugs [10]. Moreover, of the total CYP, cytochrome P450 3A4 (CYP3A4) constitutes more than 50% of total cytochrome P450 in intestine [11] and 30% of total CYP content in liver [12], [13]. Besides high amounts of CYP3A protein, intestinal enterocytes also expresses high amount of Pgp membrane transporter, and forms the first line of barrier for orally administered drugs. Additionally, these two protein (CYP3A and Pgp) shares substrate, inducer and inhibitor specificity and have coordinately regulated expression [14], [15], that might result in altered oral bioavailability of substrate drugs for these proteins when administered concomitantly [16]. For instance, St. John’s wort (*Hypericum perforatum*), Ginkgo (*Ginkgo biloba*), Grapefruit juice and garlic (*Allium sativum*) are well-studied herbs for their herb-drug interaction potential involving CYP3A4 and Pgp [17].

With increased reports of herb-drug interactions and taking into consideration the regular long time consumption of BM, potential of BM mediated herb-drug interaction needs to be evaluated for its safe and effective usage. Cytochrome P450 3A and Pgp are the major proteins of intestine and liver that are co-localized with overlap in the substrate, inhibitor, and inducer specificity. Moreover, these two proteins have been reported for co-induction and co-inhibition with St. John’s wort and grapefruit juice, respectively [18], [19]. However, no report are available regarding the modulation of CYP3A and Pgp following the BM administration, therefore, it is important to evaluate the effect of BM on CYP3A and Pgp to understand the mode of interaction. Therefore, keeping above facts in mind and considering the view that these two proteins (CYP3A and Pgp) are major protein that affects the bioavailability of orally administered drugs, the main objective of the study was to evaluate the effect of BM administration on intestinal and hepatic CYP3A and Pgp level. To examine the effect of BM mediated alteration on CYP3A4 and Pgp we measured the changes in mRNA level and functional activity of CYP3A4 and Pgp in liver and intestine of male *Sprague-Dawley* (SD) rats after BM administration for one week. In the next experiment we performed the *in-vivo* pharmacokinetic interaction study of carbamazepine (CYP 3A substrate) and digoxin (Pgp substrate) with and without BM pre-treatment in male *SD* rats to evaluate whether BM administration could alter the pharmacokinetics of CYP3A and Pgp probe drugs.

## Materials and Methods

### Reagents and Chemicals

Phenylmethanesulfonyl fluoride (PMSF), carbamazepine, carbamazepine 10,11-epoxide, rhodamine 123, tissue culture medium 199 (TC199), digoxin, bovine serum albumin (BSA) and HPLC grade were purchased from Sigma–Aldrich (St. Louis, MI, USA). Testosterone and 6β-hydroxytestosterone were purchased from the Cayman chemical company (USA). Nicotinamide adenine dinucleotide phosphate (NADPH) and dimethyl sulfoxide (DMSO), was purchased from SRL Pvt. Ltd (Mumbai, India). Ultra-pure water (18.2 M/Ωcm) was obtained from Milli-Q PLUS PF water. Bacoside enriched standardized extract of *Bacopa monniera* (Memory Sure, Lumen marketing Co., Chennai, India) was obtained from the local pharmacy shop. All other chemicals were of the highest purity commercially available or HPLC grade.

### Animals

The study was conducted in accordance with current legislation on animal experiments as per Institutional Animal Ethical Committee at CSIR-Central Drug Research Institute (IAEC approval no IAEC/2012/91). Male *Sprague-Dawley* (SD) rats of weight between 200–220 gram were provided by NLAC, CSIR-CDRI, Lucknow. Rats were housed in standard animal conditions with alternate 12 hours of light and dark cycles. Before starting to experiment, rats were acclimatized for 2–3 days. The rat dose was extrapolated to relevant clinical human dose based on surface area ratio. Rats were divided into two groups *viz.* BM pre-treated and vehicle treated control; with 5 rats in each group. Rats in pre-treated group were gavaged (16-gauge gavage needle, Kent Scientific, Torrington, CT) with BM (31 mg/kg/day) for seven days. The drug was suspended in 0.5% sodium carboxy methyl cellulose for oral administration. The control group received the same volume (1 ml) of vehicle for seven days. Animals were allowed free access to food and water but before scarification, rats were overnight fasted to reduce the intestinal content. At the end of experiment rats were euthanized with inhalation of anesthetic ether.

### RT PCR Analysis for CYP3A and Pgp Expression

Effect of BM pre-treatment on the mRNA expression of CYP3A and Pgp was evaluated using Real-time PCR. After seven days of pretreatment with BM and vehicle, rat of both groups was sacrificed with inhalation of anesthetic ether. Liver and small intestine were quickly removed, snap frozen in liquid nitrogen and stored at −80°C until RNA isolation. Total RNA from liver and intestine was isolated using TRIzol reagent (Invitrogen Life Technologies, USA) following the instruction manual. Aliquots of 2.0 µg total RNA were reverse-transcribed using a cDNA synthesis kit (Fermentas, Austin, USA). The design of sense and antisense oligonucleotide primers was based on published cDNA sequences using the Universal Probe Library (Roche Applied Sciences). Sequence for intron-spanning primer pairs of genes CYP3A1, CYP3A2, MDR1 (Multidrug resistance 1a) and GAPDH are given table 1. To ensure the specificity of primer sequences to target mRNA, each sequence was homology searched with NCBI BLAST. Primers were synthesized from Integrated DNA Technologies (USA). Real-time PCR was performed in the light cycler PCR system (Roche Molecular Biochemicals, Indianapolis, Indiana, USA) using SYBR green kit (Fermentas, Austin, USA) following manufacturer’s instructions. Fold changes in mRNA level are derived after normalizing with GAPDH mRNA level, used as internal loading control.

**Table 1:** Oligonucleotide primer used for Real-time PCR.

CYP	Gene accession no.	Forward primer sequence	Reverse primer sequence	Product size
**CYP3A1**	U09742.1	ACCCGTCTGGATTCTAAGCA	TGGAATTATTATGAGCGTTCAGC	73 nt
**CYP3A2**	L24207.1	GCAGGAGGAGATCGACAGG	CCAGGTATTCCATTTCCATCA	77 nt
**MDR1**	L15079.1	TGATGCTTTCCCCAATGC	TGTCCTCTCTCTGAAAAACTGTCA	93 nt
**GAPDH**	M17701.1	AGCTGGTCATCAATGGGAAA	ATTTGATGTTAGCGGGATCG	63 nt

### CYP3A Activity Assay

Intestinal and hepatic microsomes were prepared by methods reported earlier [20], [21] respectively, from BM treated and control groups. Briefly, for preparation of hepatic microsomes, liver was first rinsed with 0.9% sodium chloride solution and homogenized in homogenization buffer (50 mM Tris-HCl buffer, pH 7.4 containing 250 mM sucrose). Homogenate was centrifuged at 10,000×g at 4°C for 30 minutes. After centrifugation supernatant was collected and further centrifuged at 105,000×g for 60 minutes at 4°C. Pellet obtained was washed in homogenization buffer and again centrifuged at 105,000×g for 60 minutes at 4°C. Microsomal pellet thus obtained was dissolved in homogenization buffer and used for total protein estimation and enzyme assay. Similarly, for preparation of intestinal microsomes, intestine was immediately removed after sacrificing and washed with ice cold 0.9% sodium chloride to remove luminal contents. Intestine was cut open to expose the mucosal layer and villus layer of mucosa was scrapped with help of glass slide. This scrapped tissue was collected in 50 mM Tris-HCl buffer containing glycerol (20% v/v), protease inhibitor (1%) and heparin (3 U/ml) to avoid agglunitation and degradation of enzyme. Suspension was homogenized and centrifuged at 10,000×g for 20 minutes at 4°C. Supernatant was collected and centrifuged at 105,000×g for 60 minutes at 4°C. Pellet obtained was dissolved in same buffer used for homogenization excluding heparin, aliquoted and stored at −80°C until use.

Total protein content was determined by the Lowry method [21] using bovine serum albumin (BSA) as standard. Microsomes obtained were used for testosterone hydroxylation assay to assess CYP3A4 functional activity in control and BM pre-treated rats. Testosterone 6β-Hydroxylation activity was determined using the method reported earlier [22] but with slight modification. Testosterone (100 µM) was incubated with microsomes (1 and 2 mg/ml for hepatic and intestinal microsomes respectively), 10 mM MgCl_2_, in a total volume of 0.2 ml of 100 mM potassium phosphate buffer (pH 7.4). The reaction mixture was pre-incubated for 5 minutes at 37°C. The reaction was started with the addition of NADPH (2 mM) and terminated by adding 0.2 ml of acetonitrile (containing 10 µg/ml dexamethasone, internal standard) after 20 min at 37°C. Blank contains equal amounts of buffer. After centrifugation at 12,000 rpm for 15 min, supernatant was used as HPLC-UV sample. Quantification of 6β-Hydroxytestosterone formed was done from a standard curve using HPLC-UV. HPLC-UV separation was achieved on a reverse phase C18 Ultracarb 5 micron ODS (100×4.6 mm) column (phenomenex). Mobile phase consists of water (A) and acetonitrile (B) and gradient program was used for separation of analytes with conc. of B set at 30, 40, 40, 50, 80, 80 and 30% at 0, 5, 6, 7, 10, 13 and 15 min at ﬂow rate of 0.6 ml/min. Column effluent was monitored at 240 nm. Activity was expressed as nMol of product formed/min/mg. The reaction was linear with respect to incubation time and protein concentration.

### Immunoblot Assay

BM and vehicle pre-treated rats were sacrificed on the eighth day after seven days of consecutive BM treatment. Liver, kidney and intestine were quickly excised out and snap frozen in liquid nitrogen, and stored at −80°C until analysis. However, intestine before snap frozen was washed with ice cold normal saline containing 1 mM phenylmethylsulfonyl fluoride (PMSF) as protease inhibitor. Samples (Intestine, liver and kidney) were homogenized in lysis buffer (50 mM Tris containing 1% Triton X-100, pH 8) with 1 mm of PMSF and protease inhibitor cocktail (cat.no. P8340, Sigma–Aldrich, USA) at 4°C. Homogenate was centrifuged at 10,000 rpm at 4°C for 20 minutes, supernatant was collected and stored at −80°C until use. Protein in the supernatant was determined using the Lowry method [23] with BSA as standard. Fifty micrograms protein of each sample was boiled for 10 min in denaturing sample buffer (10% glycerol, 1% SDS, 1% ß-mercaptoethanol, and 0.01% bromophenol blue, 10 mM Tris-HCl; pH 6.8) and protein was separated on 8% SDS-polyacrylamide gel. Resolved proteins were electrophoretically transferred to PVDF membrane (Fermentas, Austin, USA). Membrane was blocked for nonspecific sites with 5% skimmed milk (in phosphate buffer containing 0.05% Tween 20) for 2 hours and then probed with antibodies for Pgp (1∶700; Santa Cruz Biotechnology), and then re-probed with ß-actin antibody (1∶5000; Cayman chemical company) for loading correction. Subsequently, the blots were washed three times with 0.1% Tween 20 in phosphate buffered saline (PBST) and incubated with 1∶10,000 dilution of secondary antibody (anti IgG-horseradish peroxidase conjugate) for 2 hours at room temperature. After extensive washing in PBST, substrate solution was added to the membrane, which was incubated for 5 min and exposed at room temperature. Bands were developed and visualized on X-ray film with enhanced chemiluminescence kit (ECL, Millipore), following the manufacturers protocol.

### *In-vitro* Transport of Pgp Substrate Rhodamine 123 Across BM Treated Rat Intestine

*In-vitro* transport of Rhodamine 123 (Rho123) across rat everted gut sacs were performed as described earlier [24]. Briefly, at the end of pre-treatment rats were sacrificed and ileum was taken out, washed with normal saline, and everted using a glass rod. Small intestine was cut into 4–5 cm long segments that were ligated at one end. Serosal side was filled with a Rho123 solution (25 µM rhodamine 123 in TC199 medium) and tightly ligated to create a gut sac. Immediately, this everted sac was placed in 30 ml of TC 199 medium. The solution was gassed by 95% O_2/_5% CO_2_ and maintained at 37°C throughout the experiment. Transport of Rho123 from the serosal to the mucosal side was measured by sampling 1 mL of the external medium every 10 minutes up to 80 minutes. The rate of Rho123 transport was expressed as µM or percentage secreted per minute in the mucosal compartment. In some experiments, Pgp inhibitor (verapamil 100 µM) was added in the mucosal and the serosal sides at the same concentration. This model was validated with animals pre-treated with dexamethasone (100 mg/kg/day for two days) by oral route [16].

### *In vivo* Drug Administration and Sample Collection

Animals were obtained and maintained in conditions as described earlier. Rats were divided in two groups (total 40 rats; 10 rats each group) for both probe drugs *viz.* control and BM pre-treated group. The control group received blank formulation while BM pre-treated group was treated with 31 mg/kg/day of BM consecutively for 7 days. Both, control and BM pre-treated groups were fasted overnight with free access to water. Carbamazepine (CBZ) (50 mg/kg) and digoxin (0.2 mg/kg) were administered by oral gavage using 16G needle. These drugs were used as probe drugs for CYP3A4 and Pgp respectively. However in pre-treated group probe drugs were administered only after 24 hours of last BM pre-treatment. Blood was collected from the retro - orbital plexus in heparinized tubes at designated time intervals *i.e.* 0.25, 0.5, 0.75, 1, 2, 4, 6, 8, 12 and 24 hours. Each blood sample was immediately centrifuged at 4000 rpm for 10 minutes at 4°C. Plasma was collected and stored at −80°C until use. For tissue distribution analysis of digoxin intestine, liver, kidney and brain was collected after 2 hours of digoxin administration in both groups.

### Sample Processing and Analysis of CBZ, CBZ-E and Digoxin

CBZ and its epoxy metabolite CBZ-E were extracted from 0.2 ml of plasma sample by the addition of an equal volume of ice cold acetonitrile containing internal standard phenacetin (5 µg/ml). Samples were vigorously vortexed for five minutes and centrifuged at 12,000 rpm for 20 minutes at 4°C. Supernatant thus obtained were analysed using HPLC-UV method. The HPLC system was equipped with waters binary 515 HPLC pump, waters 2998 photodiode array detector and waters 2707 Auto sampler. HPLC separation was achieved on a reverse phase C18 Syncronics 5 micron ODS (250×4.6 mm) column (Thermo scientific). Column efﬂuent was monitored at 286 nm. The mobile phase was a mixture of 10 mM ammonium acetate (pH 7.4) and acetonitrile (65∶35). Chromatography was performed at a ﬂow rate of 1.0 ml/min.

Digoxin plasma samples were analyzed using Accu Diag^Tm^ ELISA Digoxin kit (Diagnostic Automation, Inc. USA) according to manufacturer’s instructions.

### Pharmacokinetic Parameter Analysis

Pharmacokinetic parameters of probe drugs in plasma were derived from plasma concentration-time profile of probe drugs by Non-Compartmental analysis using WinNonlin Ver 5.1 (Pharsight Corporation, Mountain Veiw, CA).

### Statistical Analyses

All data were presented as mean with standard error. Statistical analyses were performed by using Student’s *t*-test (unpaired). The level of significance was set a priori at p>0.05.

## Results

### Effect of BM Pre-treatment on CYP3A mRNA Expression and Testosterone 6β-Hydroxylation Activity of Liver and Intestine

**Figure 1:**
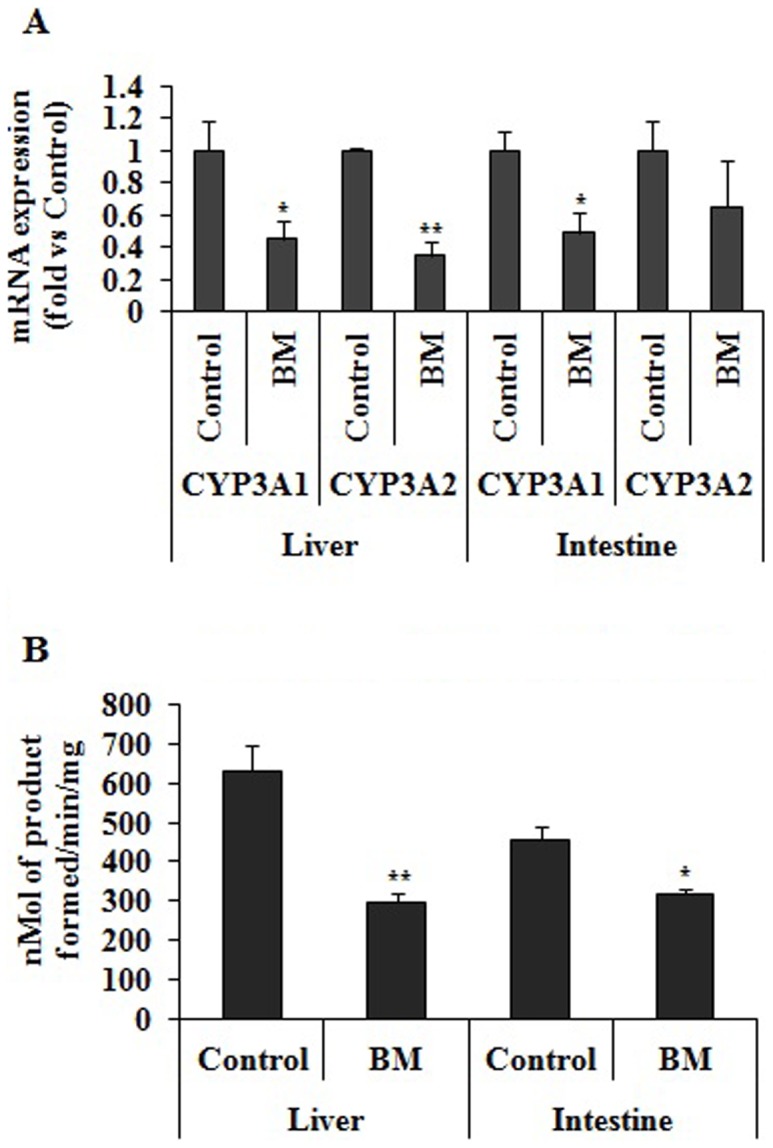
Effect of BM pretreatment on CYP3A at mRNA and functional activity level. (A) Effect of BM pretreatment on hepatic and intestinal CYP3A1 and CYP3A2 mRNA expression. BM treatment decreases the mRNA levels of CYP3A1and CYP3A2 in liver and intestine as assessed by real-time PCR as described in Materials and Methods. Fold changes in mRNA level are derived after normalizing with GAPDH mRNA level used as internal loading control (n = 3 in each group) and are indicated by numbers. BM represent rats pretreated with BM (31 mg/kg/day) for 7 days and control represents rats received same volume of vehicle. Values are expressed as mean ± S.D. All results are from 3 independent experiments with similar results; * indicates significant difference in BM pretreated rats compared to vehicle treated rats with p value <0.05. (B) Effect of BM pretreatment on functional activity of Testosterone 6β-Hydroxylation of intestine and liver. Rats were pretreated with BM (31 mg/kg/day) for 7 days and control group was treated with blank vehicle. Microsomes from intestine and liver were prepared and Testosterone 6β-Hydroxylation enzyme assay was performed as described in Materials and Methods. BM pretreatment decreases the Testosterone 6β-Hydroxylation activity in liver and intestine compared to control. BM represent rats pretreated with BM (31 mg/kg/day) for 7 days and control represents rats received same volume of vehicle Values are expressed as mean ± S.D. Asterisks* and ** significant difference in BM pretreated rats compared to vehicle treated rats with p<0.05 and p<0.001.

We assessed the status of CYP3A by quantifying the mRNA level of hepatic CYP3A1 and CYP3A2 of BM pre-treated and vehicle pre-treated groups. Hepatic CYP3A2 mRNA was significantly down regulated by 2.8 fold and significant decrease of 2.2 fold in hepatic CYP3A1 was also observed. Similarly intestinal CYP3A1 and CYP3A2 mRNA level was also decreased by 2 fold (p<0.05) and 1.5 fold (p>0.05), respectively (Figure 1A).

BM pre-treatment decreases the intestinal and hepatic testosterone 6β-hydroxylation activity by 30 and 53% respectively compared to vehicle treated control (Figure 1B).

### Effect of BM Pre-treatment on Intestinal MDR1 mRNA Expression and on Pgp Protein Levels

**Figure 2:**
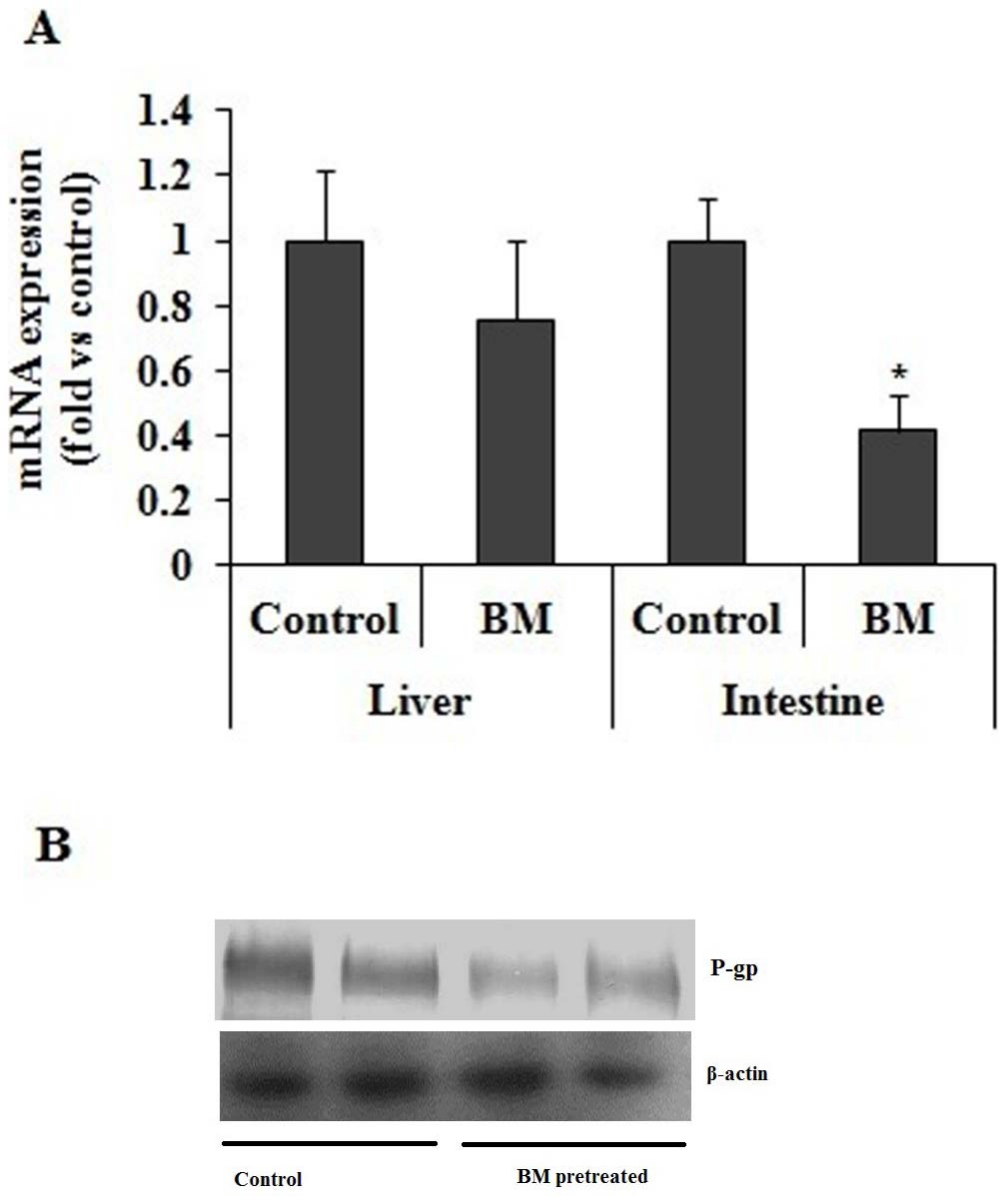
Effect of BM pretreatment on P-gp level. (A) Effect of BM pretreatment on intestine and liver MDR1 expression. BM treatment decreases the MDR1 level in intestine. Fold changes in mRNA level are derived after normalizing with GAPDH mRNA level used as internal loading control (n = 3 in each group) and are indicated by numbers. BM represent rats pretreated with BM (31 mg/kg/day) for 7 days and control represents rats received same volume of vehicle Values are expressed as mean ± S.D. all results are from 3 independent experiments with similar results; * indicates significant difference in BM pretreated rats compared to vehicle treated rats with P value <0.05. (B) Effect of BM pretreatment on P-gp protein expression. Expression of P-gp protein in intestine, liver and kidney in rats pretreated with BM (31 mg/kg/day) for 7 days and control rats treated with vehicle for same time period. Membrane for western blotting was prepared as described in Material and Methods. 50 µg of protein was separated on 7.5% SDS-PAGE. Proteins were electrotransferred to PVDF membrane and probed with anti P-gp antibody and blot was developed with ECL kit. BM represent rats pre-treated with BM (31 mg/kg/day) for 7 days and control represents rats received same volume of vehicle.

The mRNA expression profile of intestinal MDR1 of BM pre-treated and the vehicle pre-treated group was compared. In BM pre-treated group mRNA level of intestinal MDR1 was decreased by 2.5 fold compared to vehicle pre-treated group (Figure 2A). No significant difference in hepatic MDR1 mRNA was observed. Similarly weaker intensity of the bands revealed by Western blot after normalizing with beta-actin bands in the intestine of rats pre-treated with BM corresponds to a decrease in Pgp protein expression (Figure 2B). However no difference in hepatic and kidney Pgp expression was observed (data not shown).

### Effect of BM Pre-treatment on Intestinal Pgp Activity

**Figure 3:**
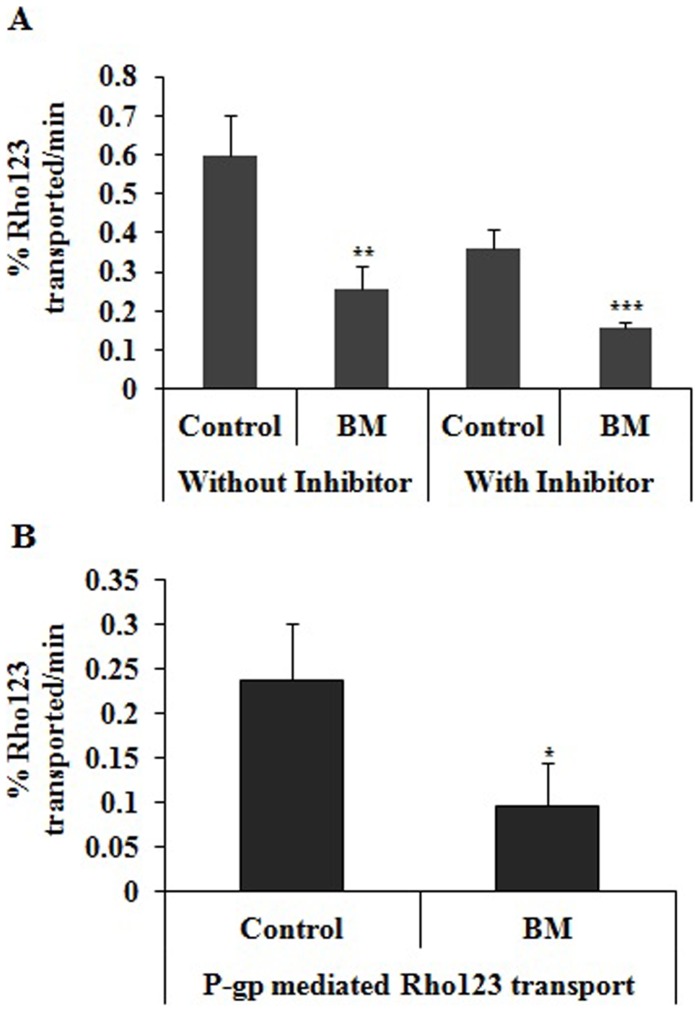
Effect of BM pretreatment on P-gp functional activity. (A) Effect of BM pretreatment on rhodamine 123 transport across intestine in BM pretreated rats and rats treated with vehicle. BM 31 mg/kg/day was given orally to rats for 7 days and on 8^th^ day rats were sacrificed. Transport of rhodamine 123 across everted gut sac from serosal to mucosal surface was assessed in absence and presence of verapamil (100 µM). (B) Effect of BM pretreatment on Pgp mediated transport of rhodamine 123 in BM pretreated rats and rats treated with vehicle. Pgp mediated transport of rhodamine 123 across everted sac was calculated as difference between transport of rhodamine in absence and presence of verapamil. BM represents rats pre-treated with BM (31 mg/kg/day) for 7 days and control represents rats received same volume of vehicle. Values are expressed as mean ± S.D. Asterisks * and ** indicates significant difference in BM pretreated rats compared to vehicle treated rats with p<0.05 and p<0.001.

Rho123 transport was measured in the absence and presence of Pgp inhibitors in everted gut sac prepared from BM pre-treated and control rats. In the absence of verapamil the transport of Rho123 was decreased by 57% in BM pre-treated rats compared with control rats. However in the presence of verapamil Rho123 transport across the everted gut sacs was significantly decreased by 60% and 62% in control and BM pre-treated rats (Figure 3A), respectively. The Pgp-mediated transport of Rho123 was significantly decreased by 60% BM pre-treated group compared with the control group (Figure 3B).

### Pharmacokinetics of CBZ and CBZ-E with and without BM Pre-treatment of Rats

**Figure 4:**
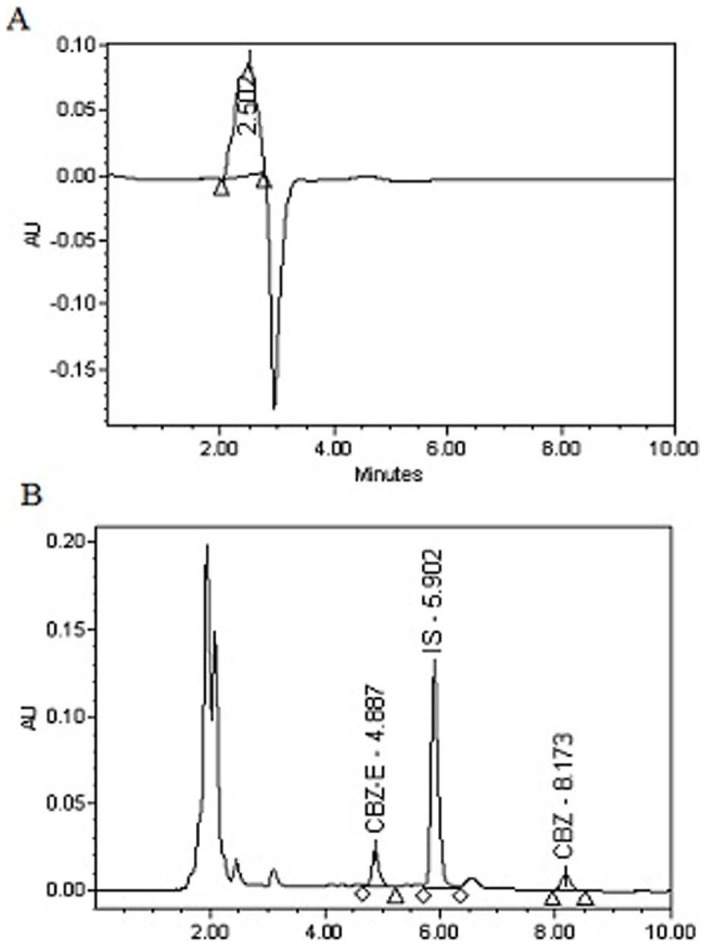
HPLC-PDA chromatograms of CYP 3A probe drug. (**A**) Chromatogram of blank plasma extracted with acetonitrile (**B**) Chromatogram of plasma spiked with CBZ and CBZ-E and extracted with phenacetin (internal standard) containing acetonitrile.

Bioanalytical method for CBZ and CBZ-E used for sample analysis was validated in terms of linearity, accuracy, precision and specificity (Figure 4). Accuracy was determined by comparing the observed concentration and spiked concentration in plasma. Similarly precision (intra-day and inter-day) was calculated at three different concentrations (Table 2). Regression equation and correlation coefficient of CBZ and CBZ-E are given in Table 3. No interference peaks were observed at the retention time of probe drug and internal standard, so confirming the specificity of bioanalytical method.

**Table 2:** Intra-day and inter-day accuracy and precision of carbamazepine and carbamazepine 10,11-epoxide in spiked plasma sample.

CBZ					CBZ-E				
Conc. (µg/mL)	% Bias Intra-day assay	% Bias Inter-day assay	% RSD Intra-day assay	% RSD Inter-day assay	Conc. (µg/mL)	% Bias Intra-day assay	% Bias Inter-day assay	% RSD Intra-day assay	% RSD Inter-day assay
**LLQC (0.078)**	10.12	9.77	3.78	10.26	LLQC (0.3)	2.89	4.91	11.49	11.19
**LQC (0.312)**	2.32	5.55	3.80	5.59	LQC (1.5)	2.14	7.97	10.10	13.28
**MQC (1.25)**	−8.60	−0.66	9.93	10.92	MQC (5)	−2.88	−2.93	11.55	11.11
**HQC (10)**	−7.51	−5.78	7.60	6.27	HQC (10)	−11.34	−3.17	12.38	6.80

**Table 3:** Regression equation and correlation coefficient of carbamazepine and carbamazepine 10,11-epoxide.

Analyte	X value	Intercept	Correlation coefficient (R^2^)
**CBZ**	0.7848±0.0350	−0.0226±0.006	0.998±0.0012
**CBZ-E**	0.0718±0.0045	0.0066±0.0076	0.996±0.0028

**Figure 5:**
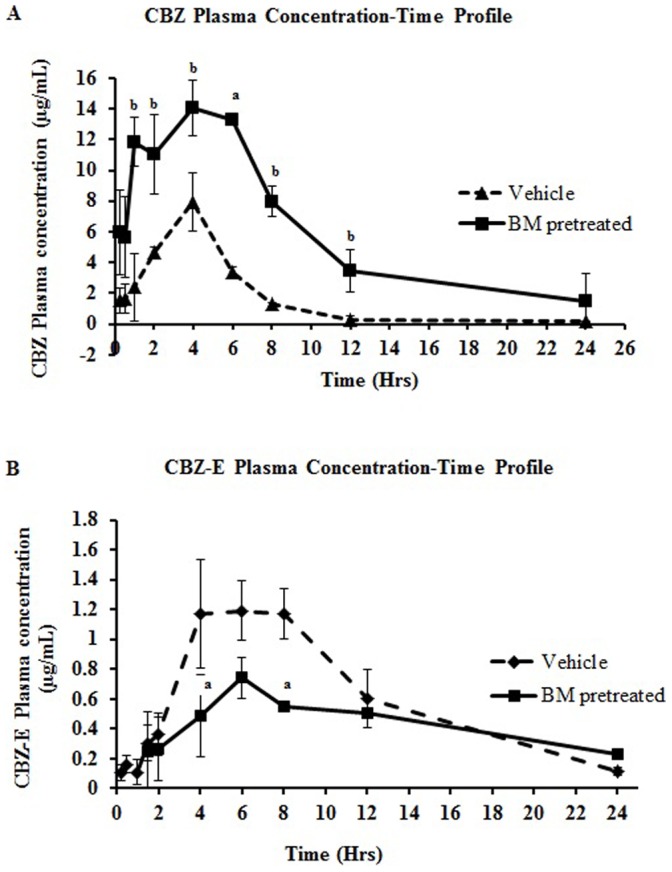
Plasma concentration time profile of CYP3A probe drug and its metabolite. (A) Time profile plasma concentration of CBZ and (B) CBZ-E in BM pretreated and control (vehicle pretreated) rats. Rats (n = 3) were orally administered with BM (31 mg/kg/day) for seven days in BM pretreated rats while control group received the same amount of vehicle. On the 8^th^ day CBZ (50 mg/kg) was given orally in both groups of rat. PK study in BM pretreated rats was performed only after 24 hours of last BM dose. Data point represents mean ± SD. Superscript *^a^* and ^b^ indicates significant difference in BM pretreated rats compared to vehicle treated rats with p<0.05 and p<0.01, respectively.

We observed the decrease in CYP3A-mediated testosterone hydroxylation activity in BM pretreated rats; therefore there might be the chance of alteration in the pharmacokinetics of carbamazepine with BM administration. Therefore, we assess the effect of BM pretreatment on the oral pharmacokinetics of carbamazepine in BM pretreated rats and compared to pharmacokinetics of carbamazepine in control rats. The plasma concentration CBZ-E and CBZ were investigated in vehicle pretreated rats and BM pretreated rats. Plasma concentration-time profile of CBZ and CBZ-E is shown in Figure 5A and 5B, respectively. The pharmacokinetics parameters of both CBZ and CBZ-E are summarized in Table 4. Concentration time profile of CBZ revealed increase in plasma concentration at each time point studied. The mean AUC and Cmax of CBZ were significantly increased by 4 and 1.8 times respectively in BM pretreated rats, compared to vehicle pretreated rats. However, CL/F was decreased significantly by 3.8 times in BM pretreated rats compared to vehicle pretreated rats. Moreover, AUC ratio (CBZ-E/CBZ) was significantly decreased by 4 times.

**Table 4:** Pharmacokinetic parameters of carbamazepine and carbamazepine 10,11-epoxide in vehicle pretreated control and BM pretreated rats.

PK parameters	CBZ		CBZ-E	
	Vehicle pretreated (Mean ± SD)	BM pretreated (Mean ± SD)	Vehicle pretreated (Mean ± SD)	BM pretreated (Mean ± SD)
**C_max_ (µg/mL)**	8.17±1.46	14.43±1.22**	1.37±0.011	0.819±0.170**
AUC_(0-∞)_ (hr*µg/mL)	40.37±3.89	168.89±65.58*	14.68±2.90	15.10±7.36
**CL/F (mL/h/kg)**	1246.50±27.32	322.88±104.04***	3.31±0.56	3.80±1.54
**MRT (hr)**	5.46±1.20	10.70±6.57	10.05±0.33	21.65±7.69*
	**Vehicle pretreated (Mean ± SD)**		**BM pretreated (Mean ± SD)**	
**AUC ratio (CBZ-E/CBZ)**	0.373±0.30		0.0879±0.0155**	

BM pretreated group were given 31 mg/kg/day for 7 days while the rats in control group received the same volume of vehicle for 7 days. After 24 hours of last treatment, rats in both group received carbamazepine (50 mg/kg). Asterisks *, **, *** indicates significant difference in BM pretreated rats compared to vehicle treated rats with p value <0.05, 0.01 and 0.001 respectively.

### Pharmacokinetics of Digoxin with and without BM Pre-treatment of Rats

**Figure 6:**
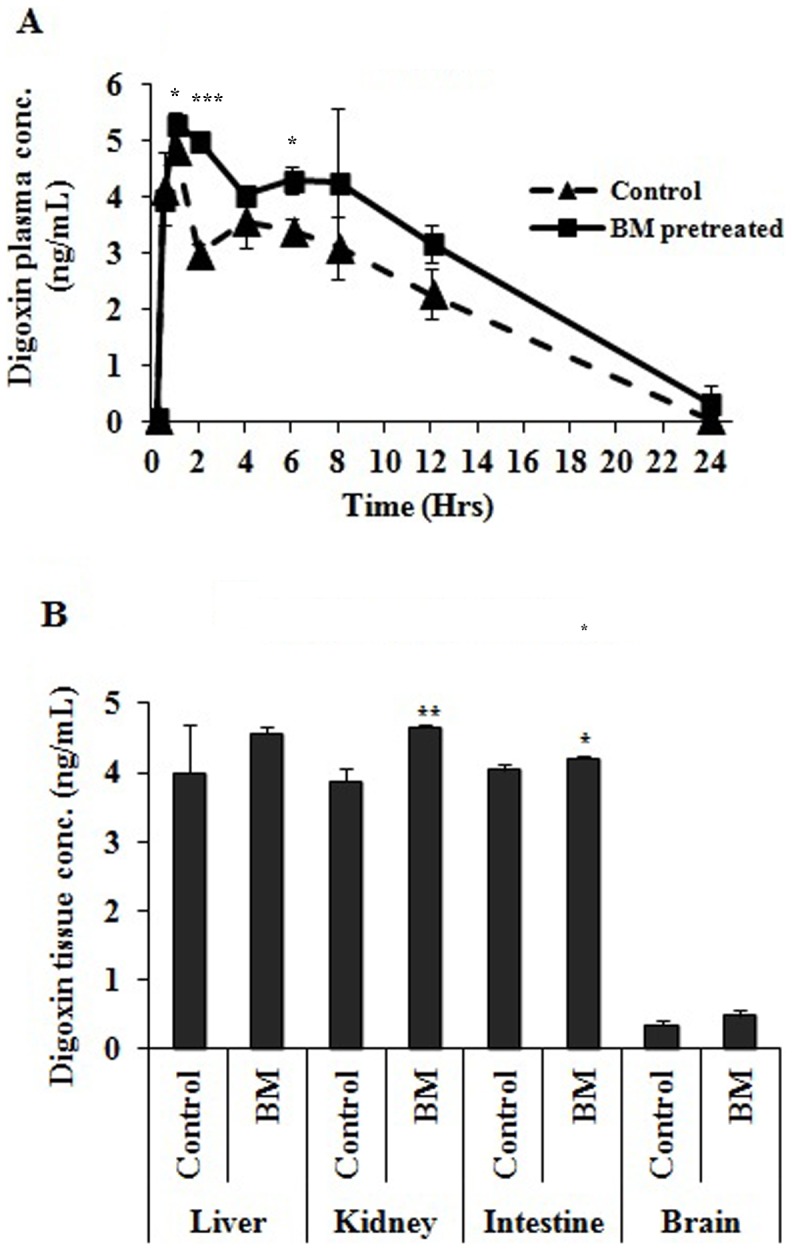
Plasma concentration time profile of P-gp probe drug. (A) Time profile of plasma concentration of digoxin in BM pretreated and control (vehicle pretreated) rats. Rats (n = 3) were orally administered with BM (31 mg/kg/day) for seven days in BM pretreated rats while control group received the same amount of vehicle. On the 8^th^ day digoxin (0.2 mg/kg) was given orally in both groups of rat. PK study in BM pretreated rats was performed only after 24 hours of last BM dose. Data point represents mean ± SD. (B) Tissue digoxin concentration in BM pretreated and control (vehicle pretreated) rats. Rats (n = 3) were orally administered with BM (31 mg/kg/day) for seven days in BM pretreated rats while control group received the same amount of vehicle. On the 8^th^ day digoxin (0.2 mg/kg) was given orally in both groups of rat. Digoxin concentration in tissue was observed after 2 hour of digoxin oral gavage. Values are expressed as mean ± S.D. Asterisks * and ** indicates significant difference in BM pretreated rats compared to vehicle treated rats with p<0.05 and p<0.001, respectively.

Similar to CBZ pharmacokinetics the plasma level of digoxin was also increased at every time point in rats pre-treated with BM compared to vehicle pre-treated group (Figure 6A). Digoxin C_max_ was significantly increased by 9% in rats with BM pre-treatment compared to vehicle treated rats. Digoxin AUC was increased by 37% in BM pre-treated rats compared to vehicle treated rats. MRT and T_1/2_ was increased by 17 and 48% respectively in BM pre-treated rats compared to vehicle treated rats. CL/F was decreased by 27% in BM pre-treated rats compared to vehicle treated rats (Table 5). Tissue digoxin concentration significantly increased by 1.3 fold in kidney and intestine. No significant difference in digoxin concentration was observed in liver and brain (Figure 6B).

**Table 5:** Pharmacokinetic parameters of digoxin in vehicle and BM pretreated rats.

PK parameters	Digoxin	
	Vehicle Pretreated	BM pretreated
C_max_ (ng/mL)	4.82±0.092	5.28±0.205*
T_1/2_ (hr)	2.92±0.322	4.33±2.032
AUC_(0-∞)_ (hr*ng/mL)	51.53±6.098	70.71±0.351*
Vd/F (mL/kg)	20.76±4.713	22.09±10.25
CL/F (mL/h/kg)	4.88±0.578	3.53±0.017*
MRT (hr)	7.56±0.0147	8.88±1.283

BM pretreated group were given 31 mg/kg/day for 7 days while the rats in control group received the same volume of vehicle for 7 days. After 24 hours of last treatment, rats in both group digoxin (0.2 mg/kg) by oral gavage for pharmacokinetics study. Asterisks * indicates significant difference in BM pretreated rats compared to vehicle treated rats with p<0.05.

## Discussion

Various reports of herb-drug interaction suggested that co-medication of herb and drugs may alter the efficacy and/or toxicity of prescribed drugs [25] and modulation in drug metabolizing enzymes and membrane transporters of the liver and intestine have been reported as an underlying mechanism involved in such herb-drug interactions [17]. CYP3A and Pgp are colocalized in intestine and have been reported for coregulated expression. The dual alteration by a single herb might result in potential herb-drug interaction [26]. Therefore, alteration of hepatic and intestinal Pgp and CYP3A with herbal consumption represents a potential mechanism that could alter the bioavailability of orally administered drugs [27]–[29]. In the present study we evaluated the effect of over the counter available herbal preparation of BM that is used for memory enhancing in children and adults on gastrointestinal CYP3A and Pgp.

Our study revealed that BM administration for seven days modulate the expression of CYP3A and Pgp in *SD* rats. It was observed that BM administration decreased the expression of CYP3A at mRNA and CYP3A-dependent testosterone hydroxylase catalytic activity in liver and intestine. Similarly, BM administration down regulates the expression of intestinal Pgp. Such co-regulation (co-induction and co-inhibition) of CYP3A and Pgp have been reported with St. john wort, grapefruit and curcumin treatment [18], [19], [30]. Additionally, no alteration in expression of kidney and liver Pgp was observed. Similar tissue specific response of Pgp has also been reported with curcumin treatment [30]. Moreover, transcription of CYP3A and Pgp is under the regulation of pregnane X receptor (PXR). Therefore, alteration in CYP3A and Pgp level with BM treatment might be attributed to its regulatory effects on PXR. Such regulation of PXR and alteration in CYP3A and Pgp level that would eventually result in altered pharmacokinetics of probe drugs has been well reported for curcumin [30].

To evaluate the effect of BM mediated alteration in CYP expression on plasma concentration of probe drug, oral pharmacokinetic herb-drug interaction with carbamazepine (CYP3A probe drug) was performed with and without BM pretreatment. The results obtained with BM pre-treatment on CBZ pharmacokinetics demonstrated an altered pharmacokinetic profile of CBZ with significant increase in C_max_ and AUC_(0-∞)_ and decrease in CL/F as compared to vehicle treated control rats. Moreover, decrease in AUC ratio of CBZ-E/CBZ and significant increase in t_1/2_ value of CBZ-E indicates reduced metabolic clearance and inhibition of CBZ to CBZ-E metabolite formation. Earlier reports suggested that CBZ is not a Pgp substrate therefore, increase in AUC of CBZ due to alteration in expression and/or activity of Pgp due to co-regulated expression of Pgp was ruled out with BM administration [31]. Rats were fasted overnight and having free access to water before the study. Furthermore, CBZ was given only after 24 hours of last BM dose. Therefore, alteration in pharmacokinetic profile of CBZ due to modulation in function of CYP3A by BM contents of previous dose was ruled out. Therefore, the underlying mechanism for changes in pharmacokinetics of CBZ and CBZ-E could be significantly attributed to down-regulation of intestinal and hepatic CYP3A protein. Furthermore, results obtained are in agreement with another study which reports the alteration in the pharmacokinetics of CBZ in rabbits with another herbal formulation “Mentat” that contains *Bacopa monniera* as one of its constituent. [32].

Similarly, effect of BM pretreatment on intestinal Pgp activity was also evaluated. Everted gut sac is the *ex-vivo* model used to assess the intestinal Pgp activity in rats using rhodamine 123 [16], [24], [33]. BM pre-treatment decreased the activity of Pgp measured with *in-vitro* everted gut sac, compared to control group. Intestinal Pgp causes drug efflux in intestinal lumen and limits the bioavailability of its substrate drugs, the down regulation of intestinal Pgp by BM was likely to contribute in the alteration of digoxin (Pgp substrate) pharmacokinetics. Therefore, to evaluate the effect of BM mediated alteration in intestinal Pgp expression on plasma concentration of probe drug, oral pharmacokinetic study with digoxin (Pgp probe drug) was performed with and without BM pretreatment. Digoxin C_max_, total AUC, MRT and T_1/2_ was significantly increased, while CL/F decreased with BM pre-treatment in comparison to control group. No significant difference was obtained in the tissue digoxin concentration of liver and brain. However significant increase in tissue digoxin concentration of intestine and kidney was observed. Digoxin study in BM pre-treated rats were performed after 24 hours of last BM treatment in order to rule out any compound mediated inhibition of Pgp, therefore, increase in the intestinal tissue digoxin concentration with BM pre-treatment could be attributed to decreased Pgp expression in intestine. Considering kidney as major organ of digoxin elimination we also evaluated the effect of BM pretreatment on kidney Pgp expression level. No significant difference in the expression of kidney Pg was observed. However, kidney tissue digoxin concentration was increased. This increased tissue concentration of digoxin in kidney may be attributed to increased plasma concentration and/or inhibition of Pgp functional activity with BM components. The modulation in Pgp activity (in-vitro everted gut sac) and pharmacokinetic parameters support the data obtained in real-time PCR and western blotting. Therefore an exact mechanism underlying behind the alteration in Pgp activity could be attributed to down regulation of Pgp level in intestine following the BM administration. Such modulation in Pgp substrate drugs with alteration in Pgp level has been observed with curcumin administration [30].

In conclusion, we report that the BM administration at clinical dose modulates the pharmacokinetics of CYP3A and Pgp substrate drugs with alteration in CYP3A and Pgp levels. The implication of study is that regular consumption of BM might influence the respective probe drug substrates. Although the major metabolic pathway for biotransformation of CBZ is common in both rat and human, but the overall biotransformation rate is significantly different between the two, with former having 10 fold higher metabolic clearance compared to later one [34]. Therefore, it is difficult to extrapolate the results obtained in rat to human. Furthermore BM consists of several components and a more detailed study of individual components, both *in-vitro* and *in-vivo,* is required in order to determine which component of this mixture is playing the dominant role. Furthermore it is imperative to evaluate BM based drug interaction with human subjects for the better understanding. It will also helpful for the prediction of clinical outcomes of undesirable side effects of BM mediated herb-drug interactions.
